# Development of a microbial protease for composting swine carcasses, optimization of its production and elucidation of its catalytic hydrolysis mechanism

**DOI:** 10.1186/s12896-022-00768-0

**Published:** 2022-11-28

**Authors:** Wei Zhai, Xintian Li, Xinran Duan, Changlong Gou, Lixia Wang, Yunhang Gao

**Affiliations:** 1 College of Animal Science and Technology, Jilin Agricultural University, Changchun, 130118 Jilin Province China; 2 College of Animal Science and Technology, Inner Mongolia University for Nationalities, Tongliao, 028000 Inner Mongolia China; 3 Northeast Institute of Geography and Agroecology, Chinese Academy of Sciences, Changchun, 130102 Jilin Province China

**Keywords:** Composting, *Serratia marcescens*, Protease, Purification, Biochemical characteristic, Catalytic hydrolysis mechanism

## Abstract

**Background:**

Dead swine carcass composting is an excellent method for harmless treatment and resource utilization of swine carcass. However, poor biodegradation ability of traditional composting results in poor harmless treatment effect. Researches report that the biodegradation ability of composting can be improved by inoculation with enzyme-producing microorganisms or by inoculation with enzyme preparations. At present, the researches on improving the efficiency of dead swine carcass composting by inoculating enzyme-producing microorganisms have been reported. However, no work has been reported on the development of enzyme preparations for dead swine carcass composting.

**Methodology:**

The protease-producing strain was isolated by casein medium, and was identified by 16 S rRNA gene sequencing. The optimal fermentation conditions for maximum protease production were gradually optimized by single factor test. The extracellular protease was purified by ammonium sulfate precipitation and Sephadex G-75 gel exclusion chromatography. The potential for composting applications of the purified protease was evaluated by characterization of its biochemical properties. And based on amino acid sequence analysis, molecular docking and inhibition test, the catalytic hydrolysis mechanism of the purified protease was elucidated.

**Results:**

In this study, a microbial protease was developed for swine carcass composting. A protease-producing strain DB1 was isolated from swine carcass compositing and identified as *Serratia marcescen*. Optimum fermentation conditions for maximum protease production were 5 g/L glucose, 5 g/L urea, 1.5 mmol/L Mg^2+^, initial pH-value 8, inoculation amount 5%, incubation temperature 30 °C and 60 h of fermentation time. The specific activity of purified protease reached 1982.77 U/mg, and molecular weight of the purified protease was 110 kDa. Optimum pH and temperature of the purified protease were 8 and 50 °C, respectively, and it had good stability at high temperature and in alkaline environments. The purified protease was a Ser/Glu/Asp triad serine protease which catalyzed substrate hydrolysis by Glu, Arg, Ser, Asp and Tyr active residues.

**Conclusions:**

In general, the microbial protease developed in this study was suitable for industrial production and has the potential to enhance composting at thermophilic stage. Moreover, the catalytic hydrolysis mechanism of the protease was further analyzed in this study.

**Supplementary Information:**

The online version contains supplementary material available at 10.1186/s12896-022-00768-0.

## Introduction

Swine carcass composting is a process of aerobic microbial fermentation, involving decomposition of organic matter and synthesis of humus [1]. Compared with other swine carcass harmless treatment methods, composting is relatively simple and inexpensive, which only needs to lay out the materials (carcass and carbon materials) and adjust the physicochemical properties (moisture content and C/N) [2]. Due to the progress of microbial fermentation and organic decomposition, the composting process went through four stages (Mesophilic, Thermophilic, Cooling and Mesophilic) [3]. Because of the high temperature and high biodegradability in the thermophilic stage, the ability of composting to kill pathogens and decompose carcass is reliable [4, 5]. Accordingly, improving the treatment capacity of thermophilic stage is a feasible strategy to enhance swine carcass composting [6]. At present, composting is recognized as an environmentally safe method of carcass disposal for routine and emergency [7, 8].

Microorganisms in compost are responsible for degradation of organic matter, although an insufficient number of indigenous microorganisms or limited enzyme production capacity may impair biodegradability [9]. However, biodegradability of compost can be improved by inoculation with enzyme preparations [10, 11] or enzyme-producing microorganisms; the latter can secrete extracellular enzymes to hydrolyze organic matter, thereby increasing compost biodegradability [12, 13]. In addition, inoculation of enzyme preparations into compost significantly hastened degradation of organic matter [14], with more direct action than adding microorganisms to produce enzymes.

As swine carcasses are primarily protein and fat, inoculating protease-producing bacteria and lipase-producing bacteria can improve degradation efficiency and improve the quality of the composting product [6]. However, there are apparently no reports on development of lipase or protease to promote compositing of swine carcasses. As swine carcasses have more protein than fate, development of a protease is the first priority.

Although animals or plants produce proteases, microorganisms are regarded as the optimal source of protease production as they are usually simple, cost-effective and highly functional [15]. Biochemical properties of microbial enzyme are determined by the separation environment of the enzyme-producing strain [7][16]. Therefore, it is necessary to select a suitable microbial protease separation environment, in accordance with the proposed microbial protease application, before selecting a microbial protease[17]. During compositing of swine carcasses, prevailing conditions include high temperature and an alkaline pH [18]. Therefore, heat and alkali resistance are critical properties for protease to promote composting.

Microorganisms with protease-producing ability are abundant in swine carcass composting [19], making the composting material an excellent resource library for isolating protease-producing microorganisms. And most of the microbial protease in compost have heat resistance and alkali resistance [20, 21]. Therefore, protease produced by bacteria isolated from swine carcass composting should be suitable for facilitating composting of swine carcasses.

Proteases can decompose proteins or peptides by catalyzing hydrolysis of peptide bonds; they all belong to group 3 of the hydrolases and subgroup 4 [22]. However, it is necessary to analyze biochemical properties to select an optimal formulation and to produce new knowledge of biochemical properties of protease.

The aim of this work was to develop a microbial protease for composting swine carcass and to analyze its application potential and catalytic hydrolysis mechanism. After screening, optimization and purification, the microbial protease had been development. And it was characterized by a series of biochemical characteristic experiments to analyze its application potential in swine carcass composting. In addition, in order to study the catalytic hydrolysis mechanism of microbial protease, the interaction between purified protease and substrate was determined by amino acid sequence analysis, homology modeling and molecular docking.

## Materials and methodology

### Sample collection and isolation of protease-producing strain

As a source of microorganisms, samples were collected from swine carcass and sawdust composting on the 10d, and immediately transported to the laboratory. The information of composting experiment used for sampling was shown in Additional file 1: Tables S1 and S2. Approximately 10 g of sample was added to a sterilized 100 mL conical flask containing 90 mL of sterilized 0.85% saline and glass beads. The conical flask was shook for 2 h at 37 °C and 120 rpm, then left to stand for 30 min, and 100 µL supernatant was removed, inoculated in casein medium (casein 4.00 g/L, Na_2_HPO_4_·12H_2_O 1.07 g/L, KH_2_PO_4_ 3.00 g/L and agar 20.00 g/L) and the culture plates incubated at 37 °C for 48 h. Protease-producing strains were screened by measuring the ratio of hydrolysis circle diameter to strain diameter. The strain with the largest ratio of hydrolysis circle diameter to strain diameter was selected as the optimal protease-producing strain, purified on Luria-Bertani (LB) medium, and assessed by Gram staining.

### Crude protease production

Pre-culture of protease-producing strain was prepared by seeding a single colony into 5 ml mineral salt medium (CH_3_COONa·3H_2_O 1.00 g/L, NH_4_Cl 1.00 g/L, NaCl 1.00 g/L, KH_2_PO_4_ 0.50 g/L, K_2_HPO_4_ 1.50 g/L, and MgSO_4_.7H_2_O 0.20 g/L; pH 7 ± 0.2), incubated at 37 °C and 120 rpm onto a rotary shaking incubator until OD_600nm_ was 1.0. Subsequently, 1% (v/v) of the pre-culture was inoculated into a 150 mL conical flask containing 100 mL mineral salt medium and the flask incubated at 37 °C and 120 rpm for 48 h. Then, the culture was centrifuged (7104 g, 4 °C for 20 min) and supernatant collected as a crude protease.

### Assay method of protease activity and protein concentration

Protease activity was determined as described [23], with protease unit (U) defined as the amount (µg) of tyrosine produced from hydrolysis of casein by 1 mL protease solution at 40 °C and pH 7.5 in 1 min [24]. Protein concentration was determined by the BCA method [25], using a commercial BCA protein assay kit (Thermo Fisher Scientific, Rockford, USA).

### Bacterial identification

Genomic identification was based on 16 S rRNA gene sequencing. Genomic DNA was purified with an Ezup Column Bacteria Genomic DNA Purification Kit (Sangon Biotech, Shanghai, China) and used as a template for amplification of 16 S rRNA, using the following primers: forward (5′-AGAGTTTGATCCTGGCTCAG-3′) and reverse (5′-GGTTACCTTGTTACGACTT-3′). Initial denaturation at 94 °C for 5 min was followed by 30 cycles at 94 °C for 30 s, 50 °C for 30 s, 72 °C for 1 min, plus final extension at 72 °C for 10 min. The amplified 16 S rRNA fragment was sliced from the 1% agarose gel, purified using a FastPure Gel DNA Extraction Mini Kit (Vazyme, Nanjing, China) and the resulting fragment submitted to Sangon Biotech (Shanghai, China) for nucleotide sequencing and comparison to the nucleotide database of NCBI using the BLAST nucleotide. A multiple sequence alignment program, CLUSTAL-W in MEGA X software, was used to align nucleotide sequences and to prepare a phylogenetic tree using the Neighbor-Joining tree approach.

### Optimization of protease production

To optimize protease production, medium composition and fermentation conditions were gradually optimized by single-factor experiments. In the optimization experiments, each treatment was repeated three times.

### Optimum carbon source

Based on the previous production method of crude proteases, sodium citrate, sucrose, glucose, maltose, CMC-Na was used to replace CH_3_COONa as carbon source, respectively. The activities of crude proteases were determined to determine the optimum carbon source. Adjust the optimal carbon source concentration to 1, 3, 5, 7, 9 g/L respectively, and determine the protease activity to determine the optimal carbon source concentration.

### Optimum nitrogen source

Based on the experiment results of optimum carbon source, Urea, (NH_4_)_2_SO_4_, NaNO_3_, yeast powder, peptone was used to replace NH_4_Cl as nitrogen source, respectively. The activities of crude proteases were determined to determine the optimum nitrogen source. Adjust the optimal nitrogen source concentration to 1, 3, 5, 7, 9 g/L respectively, and determine the protease activity to determine the optimal nitrogen source concentration.

### Optimum metal ion

Based on the experiment results of optimum nitrogen source, metal ions (Cu^2+^, Mn^2+^, Mg^2+^, Zn^2+^, Fe^3+^, Fe^2+^, Ca^2+^) were added to increase protease production, respectively. The activities of crude proteases were determined to determine the optimum metal ion. Adjust the optimal metal concentration to 0.5, 1.0, 1.5, 2.0, 2.5 mmol/L respectively, and determine the protease activity to determine the optimal metal ion concentration.

### Optimum incubation temperature

Based on the experiment results of optimum metal ion, incubation temperatures were set to 20, 25, 30, 35, 40, 45 °C. The activities of crude proteases were determined to determine the optimum incubation temperature.

### Optimum initial pH-value

Based on the experiment results of optimum incubation temperature, initial pH-values were set to 4, 5, 6, 7, 8, 9. The activities of crude proteases were determined to determine the optimum initial pH-value.

### Optimum inoculation amount

Based on the experiment results of initial pH-value, inoculation amounts were set to 1%, 3%, 5%, 7%, 9%. The activities of crude proteases were determined to determine the optimum inoculation amount.

### Optimum fermentation time

Based on the experiment results of inoculation amount, fermentation times were set to 12, 24, 36, 48, 60, 72 h. The activities of crude proteases were determined to determine the optimum fermentation time.

### Protease purification

#### Ammonium sulfate precipitation

Crude protease (20 mL) was put in a beaker that was placed in an ice bath, and ammonium sulfate powder slowly added to reach 20% saturation. Thereafter, this step was repeated, making the concentration of ammonium sulfate reach 30%, 40%, 50%, 60%, 70%, and 80% respectively. After that, the beakers were placed overnight in the refrigerator (4 °C), and then the contents centrifuged at 12,800 g for 30 min at 4 °C. Supernatants were retained, and precipitations were resuspended with 5 mL citric acid-sodium hydrogen phosphate buffer (pH 7.5). Protease activity and protein concentration in supernatant and precipitate were measured. The specific activity was determined to identify the optimum salting-out interval and to create a salting-out curve.

According to the optimum salting-out interval, 200 mL crude protease was precipitated by ammonium sulfate. The precipitation was resuspended in 100 mL buffer solution and the salt precipitation sample was dialyzed overnight using a 14 kDa MW membrane to remove ammonium sulfate. Therefore, it was concentrated to 40 mL by polyethylene glycol (PEG) 20,000.

#### Sephadex G-75 gel filtration chromatography

The protease was further purified by gel filtration chromatography, using a Sephadex G-75 chromatography column (20 × 1.6 cm). The column was pre-equilibrated with citric acid-sodium hydrogen phosphate buffer (pH 7.5). The elution was adjusted to a flow rate of 0.5 mL/min, with each 3 mL collected as a fraction. Thereafter, protein concentration and protease activities of the fractions were determined. Fractions with the highest specific activities were combined and used for subsequent studies.

#### Molecular weight determination

At successive stages of purification, molecular weights were estimated by SDS-PAGE [26]. Proteins were separated with 12% (W/V) acrylamide, and a protein marker mixture (Thermo Fisher, Shanghai, China) was applied to assess molecular weights. Gels were stained with 0.25% Coomassie blue (R-250) and de-stained in 1% acetic acid.

#### Amino acid sequence determination

The protein region in SDS-PAGE was excised and washed three times with 50% ACN/100 mm NH_4_HCO_3_ (pH 8.0) solution, vibrated for 10 min, and then the washing solution discarded. This step was repeated three times. Thereafter, the gel was cleaned with 100% ACN, and dried in a vacuum. Then, 10 mM dithiothreitol (DTT)/50 mM NH_4_HCO_3_ (pH 8.0) solution was added to the gel and incubated at 56 °C for 1 h. Thereafter, 55 mM iodoacetamide/50 mM NH_4_HCO_3_ (pH 8.0) solution was added into the gel and incubated in dark for 30 min. The gel was washed in 100% ACN, an appropriate amount of trypsin added and completely covered with 50 mM NH_4_HCO_3_ solution. The reaction system was incubated overnight at 37 °C for enzyme digestion and protein was extracted twice with 60% ACN/5% formic acid. Extracts were combined, desalted with a C18 small column and the sample frozen (-20 °C).

Mass spectrometry was done with a Thermo Q Exactive Plus system. The sample was separated by a liquid phase UltiMate 3000 RSLCnano system with nano-lift flow rate. The peptide sample was dissolved in sample buffer, loaded with an automatic injector, then combined with the C18 capture column (3 μm, 120 Ω, 100 μm × 20 mm), and eluted to the analytical column (2 μm, 120 Ω, 75 μm × 150 mm) for separation. Two mobile phases (mobile phase A: 3% dimethyl sulfoxide (DMSO), 0.1% formic acid, and 97% H_2_O; and mobile phase B: 3% DMSO, 0.1% formic acid, and 97% ACN) were used to establish the analytical gradient. The flow rate of liquid phase was \300 nL/min. In MS DDA mode analysis, each scan cycle contained one MS full scan (R = 70 K, AGC = 3e6, maxIT = 20 ms, scan range = 350–1800 m/z) and subsequent 15 MS/MS scans (R = 17.5 K, AGC = 2e5, maxIT = 100 ms). The HCD collision energy was 28, filter window for the four lever was 1.6 Da, and dynamic exclusion time for repeated ion collection was 35 s.

Mass spectrometry data generated by Q Exactive Plus were retrieved by ProteinPilot (V4.5) and the Paragon database retrieval algorithm. The screening standard of retrieval results was Unused ≥ 1.3. After deleting contaminated protein, the remaining identification information was used for subsequent analyses.

### Homology modelling and substrate docking studies

The three-dimensional structure of actin protein was downloaded from previous models in the repository of the SWISS-MODEL Web Server (https://swissmodel.expasy.org/) [27]. Amino acid sequences of purified protease were selected as templates to build a homology model via the SWISS-MODEL Web Serve, with Ramachandran plots used to evaluate model quality. All protein structures were processed in the Molecular Operating Environment (MOE 2019.1) platform, including removal of water and ions, protonation, addition of missing atoms and completion of missing groups, and protein energy minimization. Molecular docking used HDOCK software to obtain the purified protease-actin complex structure, with the purified protease and actin defined as the receptor and ligand, respectively. The protein was set to rigid, the docking contact site was set to the full surface, and the conformation generated after docking was set to 100, with the most negative energy conformation selected by the scoring function.

### Characterization of the purified protease

#### Influence of temperature on protease activity and stability

The characterization method of the influence of temperature on the activity and stability had referred to the report of Ghafoori et al. [28], and was appropriately modified in this study.

To determine the optimum temperature for protease function, a protease activity analysis was done from 30 to 80 °C. Protease activity at 40 °C was defined as 100%, and relative activity calculated. Thermal stability of the protease was investigated by pre-incubating the protease at various temperatures for 2 h, and the enzymatic reaction was conducted under standard conditions. Residual protease activity, calculated on the basis of the activity of protease without incubation, was defined as 100%.

#### Influence of pH on protease activity and stability

The characterization method of the influence of pH value on the activity and stability had referred to the report of Tito et al. and Dwivedi et al. [29, 30], and was appropriately modified in this study.

The optimal pH for protease was determined by performing the enzyme reaction in various buffers within a pH range of 3–10. The protease activity at pH 7.5 was defined as 100%, and relative activity calculated. Protease was pre-incubated in various pH values (range of 3–10) for 2 h. Subsequently, protease activity was analyzed following standard assay conditions and then residual activity was evaluated, with protease activity without incubation defined as 100%.

#### Effect of metal ions on protease activity

The characterization method of the effect of metal ions on protease activity had referred to the report of Papagianni et al. [31], and was appropriately modified in this study.

To investigate effects of metal ions on protease activity, the purified protease was pre-incubated in 1 or 5 mM of Fe^3+^, Li^+^, Fe^2+^, Mg^2+^, Ag^+^, Cu^2+^, Co^2+^, Mn^2+^, Ca^2+^and Hg^2+^ for 1 h. Protease activity was analyzed following standard assay conditions and residual activities calculated on the basis of activity of protease without metal ions being defined as 100%.

#### Effect of compounds on protease activity

The characterization method of the effect of metal ions on protease activity had referred to the report of Ghafoori et al. [28], and was appropriately modified in this study.

The influence of various compounds on protease activity was determined by treating the protease with 1 or 5 mM chemical reagents for 1 h and then measuring protease activity. Residual activity was calculated according to activity of protease without chemical reagents being defined as 100%. Compounds selected in this experiment included phenylmethylsulfonyl fluoride (PMSF), DTT, ß-mercaptoethanol, DMSO, and ethylenediaminetetraacetic acid (EDTA).

## Results and discussion

### Isolation and identification of the protease-producing strain

**Fig. 1:**
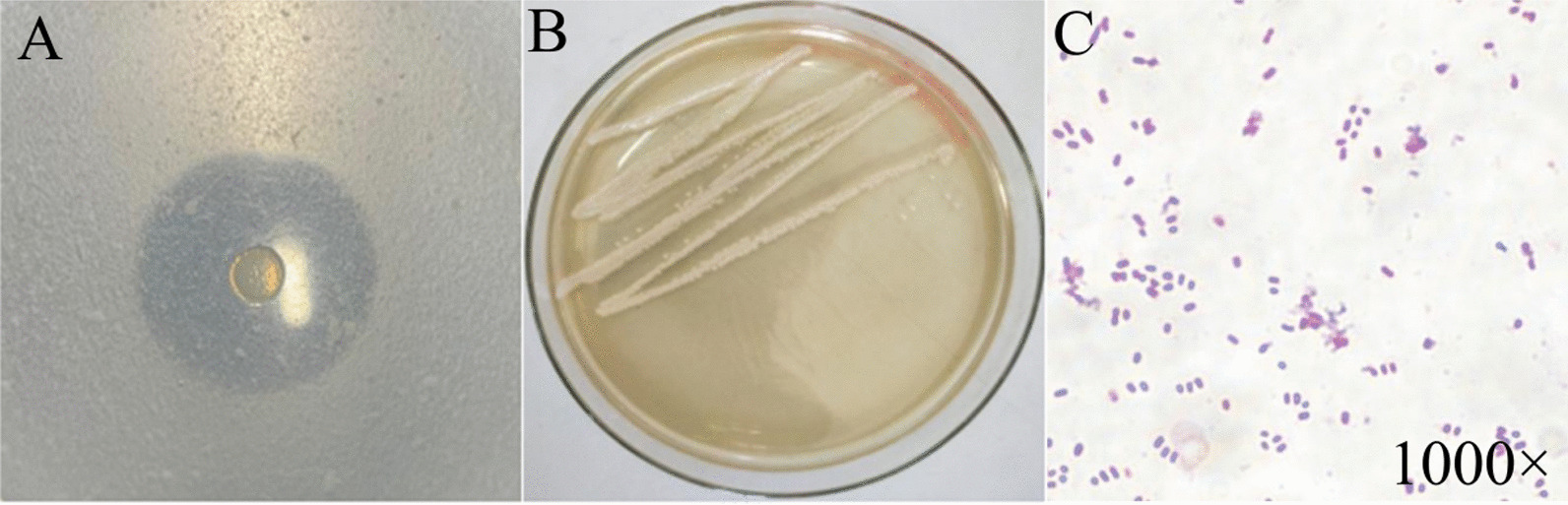
Casein hydrolysis circle (**A**), Colony (**B**) and cell morphology (**C**) of strain DB1

**Fig. 2:**
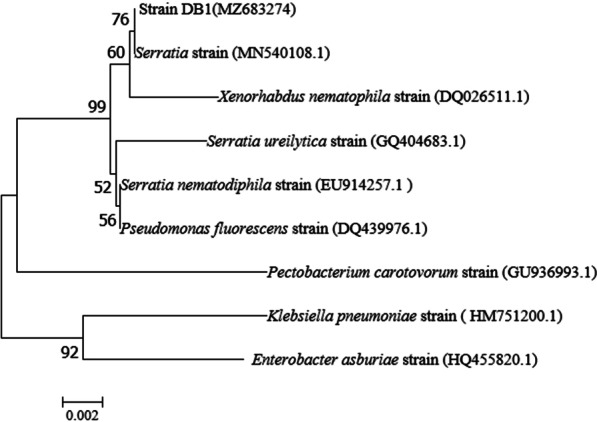
Phylogenetic analysis of DB1, on the basis of 16 S rRNA gene analysis

Among all isolates, strain DB1 had the largest ratio of hydrolysis circle diameter to strain diameter (3.93) (Fig. 1A and Additional file 1: Table S3), indicating the highest extracellular protease activity. Therefore, strain DB1 was chosen for further experimental work. Strain DB1 was white with a smooth surface on LB medium, and a short (~ 2 μm) Gram-negative rod (Fig. 1B, C). On the basis of 16 S rRNA gene sequence analysis (deposited in the GenBank database under accession number MZ683274), strain DB1 had 97% identity to *Serratia marcescens* strain MN540408.1 (Fig. 2). Therefore, strain DB1 was defined as *Serratia marcescens* sp. It is noteworthy that *Serratia marcescens* has been widely reported in chitin production, detergent production, bioremediation and hydrolase production [23–25, 32]. However, there are few reports about protease-producing *Serratia marcescens* isolated from composting. Therefore, potential differences between the protease-producing *Serratia marcescens* isolated from composting and other environments needed to be further analyzed.

### Optimization of protease production

#### Effect of medium composition on protease production

**Fig. 3:**
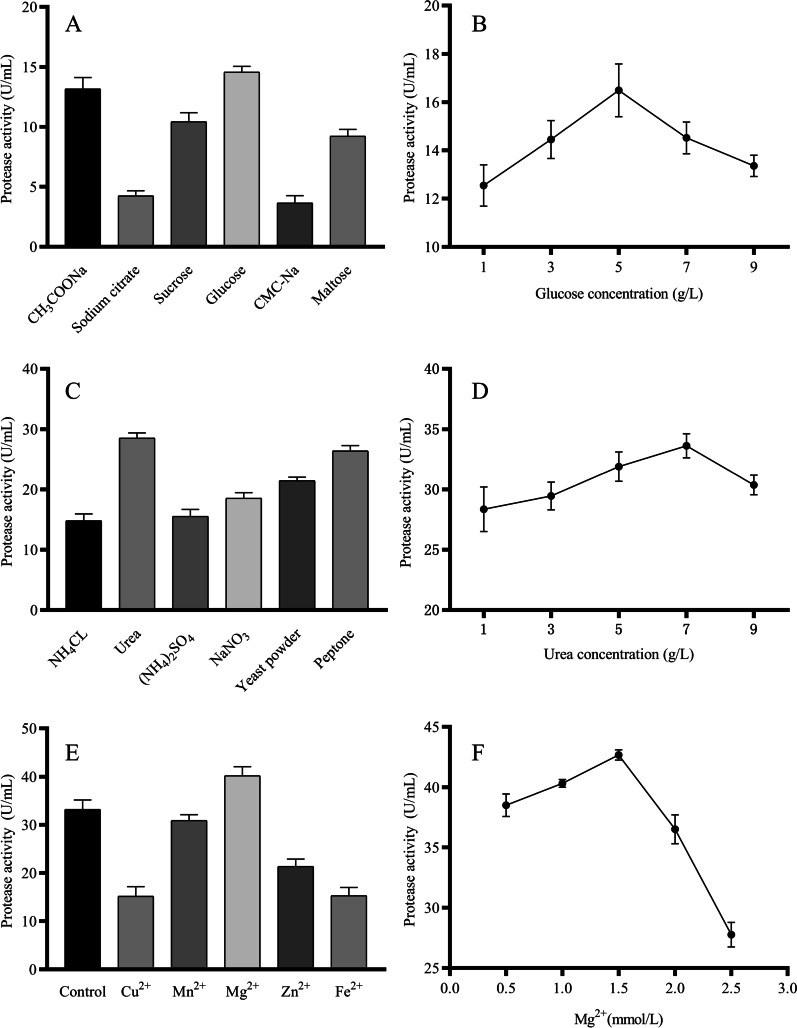
Effects of medium composition on protease production. **A** Carbon source; **B** Carbon concentration; **C** Nitrogen source; **D** Nitrogen concentration; **E** Metal ions; and **F** Metal ions concentration

Effects of medium composition on protease production are shown (Fig. 3). Among the carbon sources used, maximum protease production (14.57 U/mL) occurred in medium supplemented with glucose and CMC-Na respectively, with peak protease production (16.49 U/mL) when glucose concentration was 5 g/L. Subsequently, the carbon source was adjusted according to the experimental results.

Among the nitrogen sources studied, maximum protease production (28.52 U/mL) and least protease production (14.81 U/mL) were recorded in medium supplemented with urea and NH_4_Cl respectively. Furthermore, protease production peaked (28.52 U/mL) when the urea concentration reached 7 g/L. Therefore, the nitrogen source of the medium was adjusted to 7 g/L urea. It is worth noting that organic nitrogen sources such as yeast powder and peptone supported protease production whereas inorganic nitrogen sources such as NH_4_Cl and (NH_4_)_2_SO_4_ reduced protease production. As strain DB1 mainly used swine carcass tissues as a nitrogen source, it was presumably adapted to organic nitrogen sources.

Effects of various metal ions on protease production were shown in Fig. 3E. Although Mn^2+^ had no significant effect on protease production, Mg^2+^ increased protease production and all other metal ions reduced protease production. Metal ions participated in many biochemical and physiological processes in cells by acting as coenzyme factors or neutralizing electrostatic force in cells [33]. Due to the formation of reactive oxygen species, some metal ions were also toxic to cells at low concentrations [34], which led to the reduction of protease production by Cu^2+^, Zn^2+^ and Fe^2+^ in this study. The maximum protease production (42.66 U/mL) was achieved when the Mg^2+^ concentration was 1.5 mmol/L, which might be because Mg^2+^ was responsible for activation of some of the biosynthetic pathways [35]. Therefore, 1.5 mmol/L Mg^2+^ was added to the medium to improve protease production.

#### Effect of fermentation conditions on protease production

**Fig. 4:**
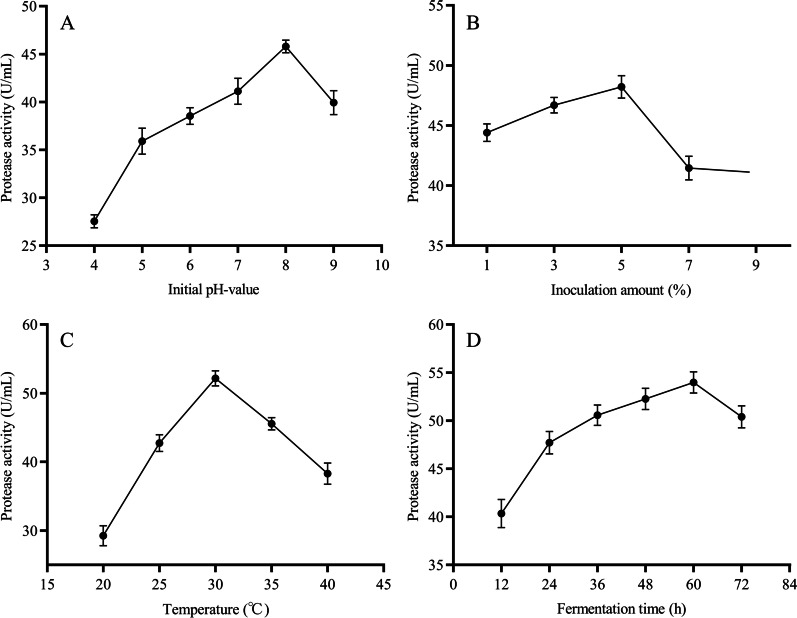
Effect of fermentation conditions on protease production. **A** Initial pH-value; **B** Inoculation amount; **C** Temperature; and **D** Fermentation time

The initial pH value affected transmembrane transport of various growth factors in bacteria, with complex impacts on production of microbial protease [25]. The optimum initial pH value for strain DB1 to produce protease was 8 (Fig. 4A), and the corresponding protease production was 45.79 U/mL.

Effects of inoculation amount on protease production are shown (Fig. 4B). In the inoculation amount range of 1–5%, protease production continuously increased as inoculation amount increased, with maximal protease production (48.21 U/mL) when the inoculation amount was 5%. However, greater inoculation amounts decreased protease production, perhaps due to consumption of nutrients for strain growth limiting nutrients for protease production [27].

Effects of incubation temperature on protease production are shown in Fig. 4C. Maximum protease production (52.17 U/mL) was achieved at 30 °C, but protease production decreased rapidly when the incubation temperature was either higher or lower. Results indicated that temperature was an influencing factor for enhanced production, because temperature could not only regulate the synthesis of protease, but also regulate the secretion of protease by changing the properties of the cell wall [36]. According to the results, the incubation temperature of strain DB1 was adjusted to 30 °C.

In the fermentation time range of 0–60 h, protease production gradually increased with increasing fermentation time, peaking (53.97 U/mL) at 60 h, whereas protease production decreased with > 60 h (Fig. 4D). Perhaps this was due to protease reacting with other compounds in the medium, resulting in denaturation and decomposition of the protease [37].

The optimum fermentation condition for *Serratia marcescens* P3 isolated from soil was 30 °C and pH7.5 [38], the optimum fermentation condition for *Serratia marcescens* KG-2-1 isolated from garbage dump was 33 °C and pH7-8 [39]. Similarly, the optimal fermentation conditions of *Serratia marcescens* DB1 was also room temperature and weak alkaline. Although the suitable fermentation conditions for different variants were similar, there were still differences among the proteases produced by different varieties (such as optimum temperature, optimum pH, stability and substrate specificity) [38–40]. As previously mentioned, the substrate specificity of the enzyme was determined by its isolation environment [16], and the enzyme isolated from compost might have some ability to resist extreme environments [20]. Therefore, whether protease produced by *Serratia marcescens* DB1 can effectively promote swine carcass composting needed to be analyzed in subsequent experiments.

In the process of industrial production, if microbial products depend on special production conditions (high temperature, low temperature, acid and alkaline, long doubling time, etc.), the cost of control process will be increased [41, 42]. The production conditions for *Serratia marcescens* DB1 were mild (moderate temperature 30 °C, alkalescence pH8) and optimum fermentation time was only 60 h. And these production conditions made it easy to control, and the control process cost was low. Overall, *Serratia marcescens* DB1 was entirely suitable for industrial production of microbial protease.

#### Purification of protease

**Fig. 5:**
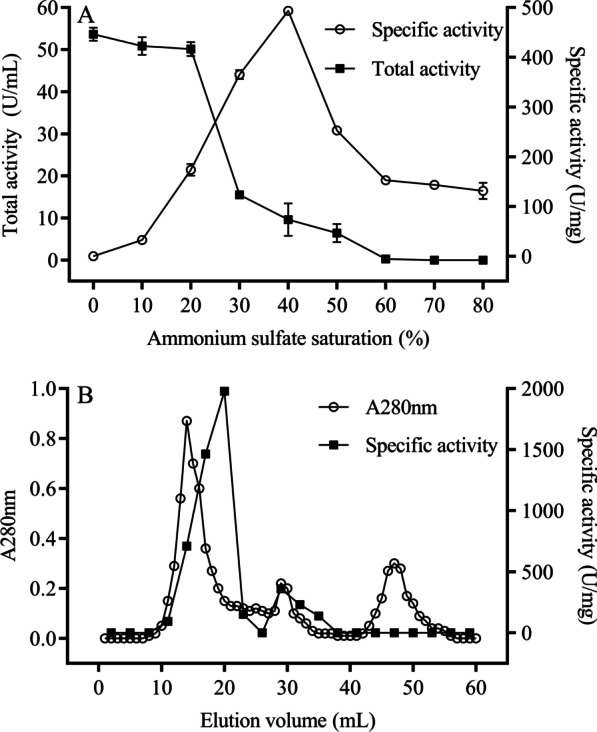
Purification of protease. **A** Ammonium sulfate precipitation; and **B** Sephadex G-75 gel exclusion chromatography

Results of purification of protease by ammonium sulfate precipitation are shown in Fig. 5A. In the ammonium sulfate saturation range of 20–40%, with an increase in ammonium sulfate saturation, protease activity in supernatant gradually decreased, and specific activity of protease in precipitation gradually increased, indicating protease was gradually transferred from the supernatant to the precipitate. However, when ammonium sulfate saturation reached 40%, specific activity of protease was maximum. Furthermore, in the range of ammonium sulfate saturation 40–80%, specific activity of protease decreased as ammonium sulfate saturation increased, indicating impurity proteins began to precipitate. Therefore, a 20–40% ammonium sulfate saturation was use to salt out the crude protease.

The protease was further purified by Sephadex G-75 gel filtration chromatography. Three protein peaks were observed during elution from the Sephadex G-75 gel column (Fig. 5B). The highest specific protease activity was detected in the first peak (10–25 mL), so it was selected as the purified protease for subsequent experiments.

After two purification steps, the specific activity of protease increased from 42.77 to 1982.77 U/mg, and the protease was purified 46.37 fold (Table 1). Furthermore, the specific activity and purification fold of purified protease in this study were higher than purification work of protease from *Serratia marcescens* reported by Anusha et al. (1033 U/mg, 21.08 fold) [43], indicating that our purified protease had potential for use in biotechnology.

**Table 1:** Summary of protease purification

Purification steps	Total activity (U)	Total protein (mg)	Specific activity (U/mg)	Purification (fold)
Crude protease	10794.00	252.37	42.77	1.00
(NH_4_)_2_SO_4_ precipitation (20–40%)	7544.35	15.30	493.09	11.52
Sephadex G-75	3271.58	1.65	1982.77	46.37

#### Identification of purified protease

**Fig. 6:**
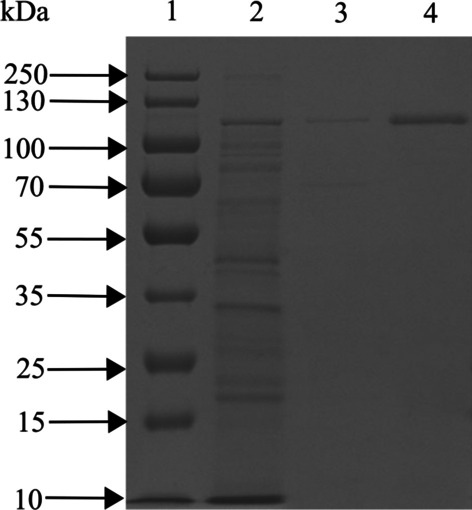
SDS-PAGE results of proteases at various purification steps (In order to improve the clarity and conciseness of the presentation, this gel was the cropped Additional file 1: Fig. S3). Line 1: Marker; Line 2: Culture supernatant; Line 3: (NH_4_)_2_SO_4_ precipitation (20–40%); and Line 4: Sephadex G-75

At each purification step, SDS-PAGE was used to determine proteases, with results shown in Fig. 6. The purified protease had a unique protein band of ~ 110 kDa in SDS-PAGE, which confirmed it had high purity. Furthermore, LC-MS/MS was used to determine amino acid sequence of the purified protease (Additional file 1: Table S4), and Table 2 has sequences of the three proteins with the highest unused (protein score values). Among them, the theoretical molecular weight of the peptides which corresponded to protein K7X482 was 112.922 kDa, consistent with the purified protease in SDS-PAGE. Moreover, the unused of protein K7X482 was much higher than other proteins. Therefore, peptides that corresponded to protein K7X482 could be considered peptides of purified protease (Additional file 1: Fig. S1). Furthermore, the theoretical amino acid sequence of the purified protease was obtained by matching sequences of these peptides in the Uniprot database (https://www.uniprot.org/) (Additional file 1: Fig. S2).

Based on a BLASTp search against UniprotKB reference proteomes plus Swiss-Prot database, the purified protease had 96.4% identity with a serine protease (protein ID: P09489) (Additional file 1: Fig. S2). Therefore, our purified protease was classified as a serine protease, named for the active hydroxyl group of the Ser residue in its catalytic center. This group active hydroxyl can act as an electron donor for the first step of peptide hydrolysis, involving activation of a nucleophile, polarization of the peptide carbonyl, and stabilization of the tetrahedral intermediate [44]. However, differences in catalytic hydrolysis mechanisms can cause great differences in biochemical properties of various serine proteases [45]. Therefore, subsequent experiments are needed to characterize the catalytic hydrolysis mechanism of purified protease.

**Table 2:** Peptide sequence of purified protease identified by LC-MS/MS against the UNIPROT protein database

Sequence	Master protein accession	Theoretical Mw (kDa)	Peptides (95%)	Unused
SQNLLSQNEAQSLLGNK	K7X482	112.922	50	45.56
TIHDILVNPGSGNNAVR	A0A656VJZ5	65.323	8	9.69
NLTASANNQLLTYGAK	A0A656VDJ2	211.911	3	8.07

#### Homology modelling and substrate docking studies

Actin, the second most abundant protein in skeletal muscle, participates in more protein-protein interactions than any known protein [46]. *Serratia marcescens* DB1 was isolated from swine carcass composting, and animal protein was the main substrate for its protease. Elucidating interactions between purified protease and actin could explain catalytic hydrolysis mechanism of the purified protease. Therefore, homology modeling and molecular docking methods were used to evaluate interactions between purified protease and actin.

**Fig. 7:**
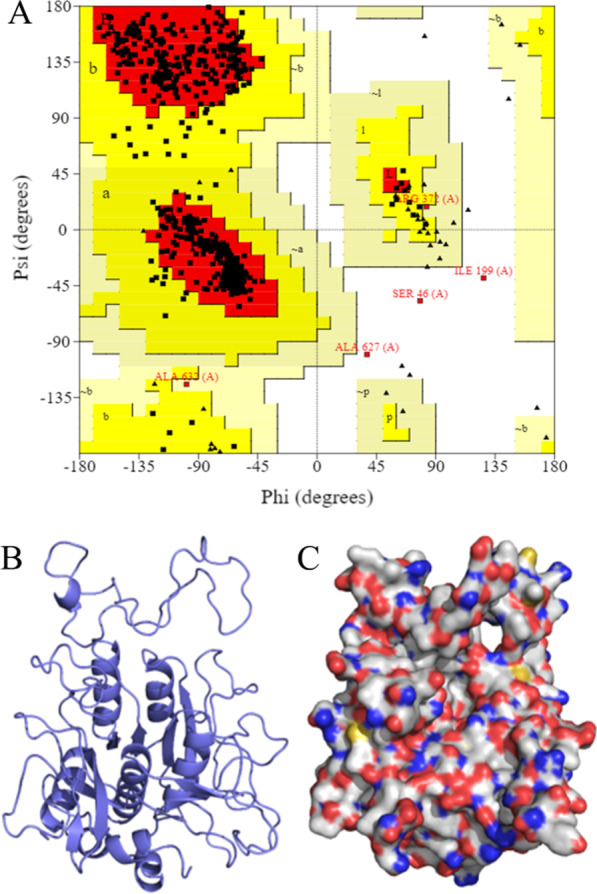
Ramachandran plot and three-dimensional structure model of purified protease. **A** The Ramachandran plot (red, yellow, light brown and white areas represent reliable, permissible, generous permissible and impermissible areas, respectively). **B** Protein backbone of purified protease. **C** Electrostatic surface of purified protease (blue represents positive, red represents negative)

Based on the Ramachandran plot (Fig. 7A), the purified protease model was composed of 723 amino acids, the number of residues in the reliable range was 671, accounting for 92.8% of total residues, and 47 residues in the permissible range, accounting for 6.5% of total residues. Residues in the reliable range and permissible range accounted for 99.6%, indicating that the model was available. The three-dimensional structure model of the purified protease is shown in Fig. 7B, C.

**Fig. 8:**
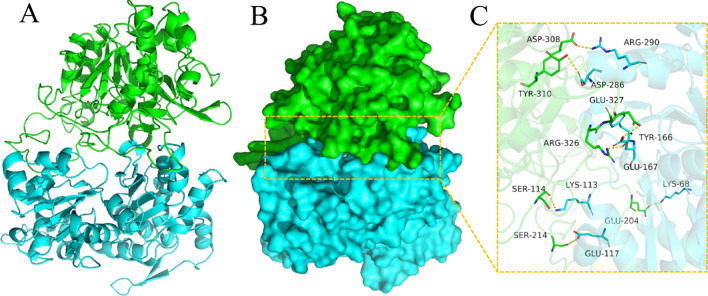
The binding mode of the complex purified protease with actin. **A** The backbone of protein was rendered as a tube and colored in green (purified) and blue (Actin). **B** Purified protease and actin protein were rendered by the surface. **C** The detail binding mode of purified protease with actin. A yellow dash represents a hydrogen bond or salt bridge

Molecular docking results of our purified protease with actin are shown in Table 3. There were many interactions between contact residues of these two proteins, such as salt bridges, hydrogen bonds, and hydrophobic interactions, which could stabilize the purified protease and actin complex. In addition, the purified protease bound to actin mainly through the following sites: Glu327-Glu167, Arg326-Tyr166, Ser114-Lys133, Ser214-Glu117, Glu204-Lys68 and Asp308-Arg290, Tyr310-Asp286 (Fig. 8) (Additional file 1: Fig. S4). Based on molecular docking results of purified protease and actin, the active center of purified protease had the following amino acid residues: Glu, Arg, Ser, Asp and Tyr. Unlike a classical Ser/His/Asp catalytic triad, this purified protease caused catalysis using a Ser/Glu/Asp triad was similar to results reported by Wlodawer et al. [47]. The Ser act as the catalytic and Glu/Asp act as the general base in catalysis. In addition, our purified protease also contained active Tyr and Arg residues, which may also affect its biochemical properties.

**Table 3:** Docking results of two target proteins (purified protease and actin)

Protein1	Protein2	Binding energy (kcal/mol)	Contact sites (protein1)	Contact sites (protein2)	Combination type
Purified protease	Actin	−259.99	Asp308 Tyr310 Glu327 Arg326 Ser114 Glu204 Ser214	Arg290 Asp286 Tyr166 Glu167 Lys68 Lys113 Glu117	Salt bridge Hydrogen bond Hydrophobic interaction

### Characterization of the purified protease

#### Effects of pH and temperature on purified protease activity and stability

**Fig. 9:**
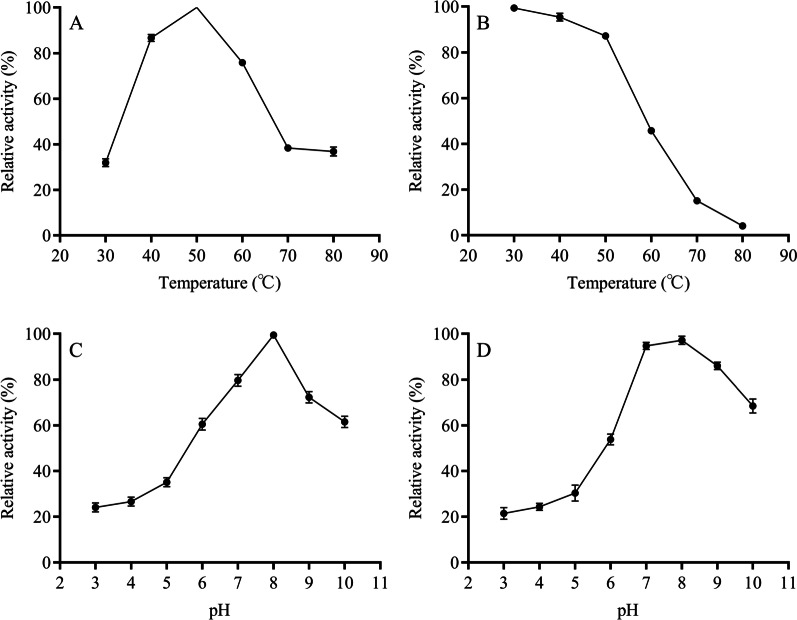
Effect of temperature on purified protease activity (**A**) and stability (**B**) and effects of pH on purified protease activity (**C**) and stability (**D**)

Effects of temperature on the purified protease activity and stability are shown in Fig. 9A and B. The purified protease activity maintained > 80% relative activity in the range from 30 to 80 °C, whereas purified protease activity was maximum at 50 °C. Furthermore, the protease was stable at a temperature range from 30 to 60 °C with more than relative activity of 80%. Gomes et al. reported a serine protease from *Mucor subtilissimus* URM 4133, with optimum temperature at 45 °C and stability > 80% (40 °C/2 h) [48]. Compared to that, the serine protease purified in this study had a higher optimum temperature and thermal stability.

The purified protease maintained > 80% of relative activity at the pH range of 7–9 and the optimal pH of purified protease was 8 (Fig. 9C), indicating the purified protease was alkaline protease. After 2 h of pre-incubation at pH range from 7 to 9, the purified protease still retained > 80% relative activity (Fig. 9D). In general, the optimal pH for Ser/Glu/Asp triad serine protease is generally acidic [45]. However, Ser/Glu/Asp triad serine protease purified in this study had an optimal pH of alkaline, due to the presence of the alkaline residue Arg in the active center of the protease (Fig. 8). Furthermore, an alkaline environment (typical of pig carcass composting) favors the action of the alkaline residue.

The performance of protease application can be influenced by many environmental factors, including pH, ionic strength, and temperature [49]. Therefore, evaluation of a protease needs to include its activity and stability in potential application environment [50]. During the main phase of swine carcass composting, temperature could be > 50 °C for at least 10 days and pH between 7 and 9 [6]. Corresponding to the previous swine carcass composting results in Additional file 1: Table S2 and in previous reports [6, 51], optimum conditions of the protease (optimum temperature, optimum pH, temperature stability and pH stability) were similar to the environmental conditions in the thermophilic stage (10d). Indicating that the protease had the potential to enhance the biodegradability of composting by applying at thermophilic stage. Furthermore, the microbial protease production conditions in this study were mild and suitable for industrial production (Figs. 3 and 4). In conclusion, these results suggested a potential application scheme of microbial protease. In real applications, this protease will be first industrially produced and then applied to thermophilic stage of swine carcass composting.

#### Effects of metal ions on purified protease activity

Effects of metal ions on the activity of purified protease are shown in Table 4. 5 mM Mn^2+^ promoted protease activity, which was similar to the report of Sharma et al. [49]. This was because some metal ions play an important role in maintaining the active conformation of the protease [52]. Moreover, 1 mM Mg^2+^, Mn^2+^, or Ca^2+^ had no significant effect on the protease activity. And 5 mM Fe^3+^, Li^+^, Fe^2+^, Mg^2+^, Ag^+^, Cu^2+^, Co^2+^, Ca^2+^ and Hg^2+^ inhibited protease activity, which was similar to serine proteases reported by Matkawala et al. and Silva et al. [53, 54]. It could be seen that different metal ions have different activation and inhibition effects on protease activity, and suitable metal ions will be beneficial in the application of protease [55]. These results indicated that 5 mM Mn^2+^ could be added to improve protease activity in the application of protease.

**Table 4:** Effects of metal ions on protease

Metal ion	Relative activity (%)	
	1 mM	5 mM
Fe^3+^	76.78 ± 1.89	12.36 ± 0.86
Li^+^	93.29 ± 1.56	88.25 ± 2.12
Fe^2+^	73.50 ± 2.36	43.46 ± 3.48
Mg^2+^	96.97 ± 1.70	81.82 ± 2.30
Ag^+^	72.56 ± 1.62	12.31 ± 1.87
Cu^2+^	42.74 ± 3.14	17.33 ± 3.08
Co^2+^	70.82 ± 0.43	27.64 ± 0.68
Mn^2+^	99.54 ± 2.91	155.87 ± 1.78
Ca^2+^	96.06 ± 2.30	45.30 ± 5.10
Hg^2+^	24.36 ± 1.10	11.28 ± 0.52

#### Effects of compounds on purified protease activity

Compounds can be used to study active residues of enzymes or to classify enzymes [56]. Effects of compounds on protease activity are shown in Table 5. It was noteworthy that PMSF (serine inhibitor) could completely inhibit protease activity, indicating that the purified protease was a serine protease. Furthermore, this also confirmed the presence of the Ser active residue in the protease active center, as suggested in Fig. 8.

**Table 5:** Effects of compounds on protease

Compounds	Relative activity (%)	
	1 mM	5 mM
PMSF	28.26 ± 0.03	3.54 ± 0.01
DTT	114.99 ± 0.01	139.53 ± 3.20
EDTA	95.99 ± 2.94	87.88 ± 2.42
ß-mercaptoethanol	108.66 ± 3.73	129.64 ± 5.64
DMSO	77.81 ± 5.21	69.35 ± 3.14

Among the active residues of purified protease, Arg, Asp and Tyr are all hydrophilic amino acids (Fig. 8). The purified protease was inhibited (77.81 and 69.35% relative activities) by 1 or 5 mM DMSO, respectively; this compound reduces enzyme activity by breaking the hydrogen bond between the enzyme and water. Furthermore, this also indicated that the hydrophilic amino acid residues of the active center are important in protease activity.

Moreover, protease activity decreased to 95.99 and 87.88% in the presence of 1 and 5 mM EDTA (metal ionic chelating agent), indicating some metal ions contribute to maintenance of purified protease activity, consistent with the speculation in Table 4. Finally, DTT and ß-mercaptoethanol (thiol protecting agents) improved activity, indicating thiol dependence.

## Conclusion

In this study, a protease-producing *Serratia marcescens* DB1 was isolated from swine carcass composting. Optimum fermentation conditions of *Serratia marcescens* DB1were mild, indicating that its production cost was low and it was suitable for industrial production.

Corresponding to the physicochemical properties of swine carcass composting, the purified protease had high activity and stability in the environment similar to the thermophilic stage (alkaline and high temperature). Therefore, the protease developed in this study had the potential to enhance composting at thermophilic stage. Moreover, the catalytic hydrolysis mechanism of the protease was further analyzed by studying the interaction between protease and substrate. Furthermore, as the present work focused on protease development and usability, follow-up studies should be conducted in at least laboratory-scale trials or ideally composting trials to evaluate effects of protease application.

## Supplementary Information

**Additional file 1:** Data generated in research.

## Data Availability

The datasets generated during the current study are available in the [NCBI, MN540408.1] repository. The peptide information datasets are available in the supplementary materials (Additional file 1: Table S1). The datasets for protein analysis are downloaded from the the [Uniprot, K7X482], and [Swiss-model, K7X482] repository.

## References

[1] MR M (2015). Using broiler litter and swine manure lagoon effluent in sawdust-based swine mortality composts: effects on nutrients, bacteria, and gaseous emissions. Sci Total Environ.

[2] Gwyther CL, Prysor Williams A, Golyshin PN, Edwards-Jones G, Jones DL (2011). The environmental and biosecurity characteristics of livestock carcass disposal methods: a review. Waste Manag.

[3] Costa T, Akdeniz N (2019). A review of the animal disease outbreaks and biosecure animal mortality composting systems. Waste Manag.

[4] Pepin B, Williams T, Polson D, Gauger P, Dee S (2021). Survival of swine pathogens in compost formed from preprocessed carcasses. Transbound Emerg Dis.

[5] Chen Y, Li X, Li S, Xu Y (2021). Effect of C/N ration on disposal of pig carcass by co-composting with swine manure: experiment at laboratory scale. Environ Technol.

[6] Yang X, Hu Q, Han Z, Ruan X, Jiang S, Chai J, Zheng R (2018). Effects of exogenous microbial inoculum on the structure and dynamics of bacterial communities in swine carcass composting. Can J Microbiol.

[7] Guan J, Chan M, Grenier C, Brooks BW, Spencer JL, Kranendonk C, Copps J, Clavijo A (2010). Degradation of foot-and-mouth disease virus during composting of infected pig carcasses. Can J Vet Res.

[8] KG W (2007). The biosecurity of on-farm mortality composting. J Appl Microbiol.

[9] Xi B, He X, Dang Q, Yang T, Li M, Wang X, Li D, Tang J (2015). Effect of multi-stage inoculation on the bacterial and fungal community structure during organic municipal solid wastes composting. Bioresour Technol.

[10] Gautam S, Bundela P, Pandey A, Jamaluddin, Awasthi M, Sarsaiya S (2012). Diversity of cellulolytic microbes and the biodegradation of municipal solid waste by a potential strain. Int J Microbiol.

[11] Jiang J, Wang Y, Yu D, Yao X, Han J, Cheng R, Cui H, Yan G, Zhang X, Zhu G (2021). Garbage enzymes effectively regulated the succession of enzymatic activities and the bacterial community during sewage sludge composting. Bioresour Technol.

[12] Zhao Y, Zhao Y, Zhang Z, Wei Y, Wang H, Lu Q, Li Y, Wei Z (2017). Effect of thermo-tolerant actinomycetes inoculation on cellulose degradation and the formation of humic substances during composting. Waste Manag.

[13] Hashimoto H, Iwaasa T, Yokotsuka T (1972). Thermostable acid protease produced by Penicillium duponti K1014, a true thermophilic fungus newly isolated from compost. Appl Microbiol.

[14] Negi S, Mandpe A, Hussain A, Kumar S (2020). Collegial effect of maggots larvae and garbage enzyme in rapid composting of food waste with wheat straw or biomass waste. J Clean Prod.

[15] Laxman RS, Sonawane AP, More SV, Rao BS, Rele MV, Jogdand VV, Deshpande VV, Rao MB (2005). Optimization and scale up of production of alkaline protease from Conidiobolus coronatus. Process Biochem.

[16] López MJ, Jurado MM, López-González JA, Estrella-González MJ, Martínez-Gallardo MR, Toribio A, Suárez-Estrella F (2021). Characterization of thermophilic lignocellulolytic microorganisms in composting. Front Microbiol.

[17] Lario LD, Pillaca-Pullo OS, Sette LD, Converti A, Casati P, Spampinato C, Pessoa A (2020). Optimization of protease production and sequence analysis of the purified enzyme from the cold adapted yeast *Rhodotorula mucilaginosa* CBMAI 1528. Biotechnol Rep.

[18] Guidoni L, Martins G, Guevara M, Brandalise J, Lucia T, Gerber M, Corrêa L, Corrêa É (2021). Full-scale composting of different mixtures with meal from Dead Pigs: process monitoring, compost quality and toxicity. Waste Biomass Valorization.

[19] Yang X, Han Z, Ruan X, Chai J, Jiang S, Zheng R (2019). Composting swine carcasses with nitrogen transformation microbial strains: succession of microbial community and nitrogen functional genes. Sci Total Environ.

[20] Lu X, Yang Y, Hong C, Zhu W, Yao Y, Zhu F, Hong L, Wang W (2022). Optimization of vegetable waste composting and the exploration of microbial mechanisms related to fungal communities during composting. J Environ Manage.

[21] Ma C, Hu B, Wei M-B, Zhao J-H, Zhang H-Z (2019). Influence of matured compost inoculation on sewage sludge composting: enzyme activity, bacterial and fungal community succession. Bioresour Technol.

[22] Rao MB, Tanksale AM, Ghatge MS, Deshpande VV (1998). Molecular and biotechnological aspects of microbial proteases. Microbiol Mol Biol Rev.

[23] Araújo H, Andrade R, Montero-Rodríguez D, Rubio-Ribeaux D, Alves da Silva C, Campos-Takaki G (2019). Sustainable biosurfactant produced by Serratia marcescens UCP 1549 and its suitability for agricultural and marine bioremediation applications. Microb Cell Fact.

[24] Chen Y, Zhu Q, Dong X, Huang W, Du C, Lu D (2019). How Serratia marcescens HB-4 absorbs cadmium and its implication on phytoremediation. Ecotoxicol Environ Saf.

[25] Jupatanakul N, Pengon J, Selisana S, Choksawangkarn W, Jaito N, Saeung A, Bunyong R, Posayapisit N, Thammatinna K, Kalpongnukul N (2020). *Serratia marcescens* secretes proteases and chitinases with larvicidal activity against *Anopheles dirus*. Acta Trop.

[26] Jyoti V, Sangeeta P (2019). Characterization of partially purified alkaline protease secreted by halophilic bacterium *Citricoccus* sp. isolated from agricultural soil of northern India. Biocatal Agric Biotechnol.

[27] Abol-Fotouh D, AlHagar O, Hassan M (2021). Optimization, purification, and biochemical characterization of thermoalkaliphilic lipase from a novel *Geobacillus stearothermophilus* FMR12 for detergent formulations. Int J Biol Macromol.

[28] Ghafoori H, Askari M, Sarikhan S (2016). Purification and characterization of an extracellular haloalkaline serine protease from the moderately halophilic bacterium, *Bacillus iranensis* (X5B). Extremophiles.

[29] Tito FR, Pepe A, Tonon CV, Daleo GR, Guevara MG (2020). Determination and characterisation of milk-clotting activity of two Solanum tuberosum aspartic proteases (StAPs). Int Dairy J.

[30] Dwivedi P, Sharma AK, Singh SP (2021). Biochemical properties and repression studies of an alkaline serine protease from a haloalkaliphilic actinomycete, *Nocardiopsis dassonvillei* subsp. albirubida OK-14. Biocatal Agric Biotechnol.

[31] Papagianni M, Sergelidis D (2014). Purification and biochemical characterization of a novel alkaline protease produced by Penicillium nalgiovense. Appl Biochem Biotechnol.

[32] Amornloetwattana R, Robinson R, Soysa H, van den Berg B, Suginta W (2020). Chitoporin from Serratia marcescens: recombinant expression, purification and crystallization. Acta Crystallogr Sect F Struct Biol Commun.

[33] Mrvčić J, Butorac A, Solić E, Stanzer D, Bačun-Družina V, Cindrić M (2013). Characterization of Lactobacillus brevis L62 strain, highly tolerant to copper ions. World J Microbiol Biotechnol.

[34] Miyoshi A, Rochat T, Gratadoux J-J, Loir YL, Oliveira SC, Langella P, Azevedo V (2003). Oxidative stress in Lactococcus lactis. Genet Mol Res.

[35] Raza W, Yang X, Wu H, Huang Q, Xu Y, Shen Q (2010). Evaluation of metal ions (zn(2+), Fe(3+) and mg(2+)) effect on the production of fusaricidin-type antifungal compounds by *Paenibacillus polymyxa* SQR-21. Bioresour Technol.

[36] Anandan D, Marmer WN, Dudley RL (2007). Isolation, characterization and optimization of culture parameters for production of an alkaline protease isolated from aspergillus tamarii. J Ind Microbiol Biotechnol.

[37] Uyar F, Baysal Z (2004). Production and optimization of process parameters for alkaline protease production by a newly isolated Bacillus sp. under solid state fermentation. Process Biochem.

[38] Bach E, Sant’Anna V, Daroit DJ, Correa APF, Segalin J, Brandelli A (2012). Production, one-step purification, and characterization of a keratinolytic protease from *Serratia marcescens* P3. Process Biochem.

[39] Taneja K, Bajaj B, Kumar S (2017). Dilbaghi N Production, purification and characterization of fibrinolytic enzyme from *Serratia* sp. KG-2-1 using optimized media. 3 Biotech.

[40] Wan M-H, Wu B, Ren W, He B (2010). Screening, characterization, and cloning of a solvent-tolerant protease from *Serratia marcescens* MH6. J Microbiol Biotechnol.

[41] Contesini FJ, Melo RR, Sato HH (2018). An overview of Bacillus proteases: from production to application. Crit Rev Biotechnol.

[42] Mageswari A, Subramanian P, Chandrasekaran S, Karthikeyan S, Gothandam KM (2017). Systematic functional analysis and application of a cold-active serine protease from a novel *Chryseobacterium*sp. Food Chem.

[43] Krishnamurthy A, Belur P (2018). A novel fibrinolytic serine metalloprotease from the marine *Serratia marcescens* subsp. sakuensis: purification and characterization. Int J Biol Macromol.

[44] Burchacka E, Pięta P, Łupicka-Słowik A (2022). Recent advances in fungal serine protease inhibitors. Biomed Pharmacother.

[45] Ekici O, Paetzel M, Dalbey R (2008). Unconventional serine proteases: variations on the catalytic Ser/His/Asp triad configuration. Protein Sci.

[46] Dominguez R, Holmes KC (2011). Actin structure and function. Annual Rev Biophys.

[47] Wlodawer A, Li M, Gustchina A, Dauter Z, Uchida K, Oyama H, Goldfarb N, Dunn B, Oda K (2001). Inhibitor complexes of the Pseudomonas serine-carboxyl proteinase. Biochemistry.

[48] Gomes J, Rosa I, Nascimento T, Souza-Motta C, Gomes E, Boscolo M, Moreira K, Pintado M, da Silva R (2020). Biochemical and thermodynamic characteristics of a new serine protease from *Mucor subtilissimus* URM 4133. Biotechnol Rep.

[49] Sharma K, Kumar R, Panwar S, Kumar A (2017). Microbial alkaline proteases: optimization of production parameters and their properties. J Genetic Eng Biotechnol.

[50] Gohel S, Singh S (2018). Thermodynamics of a Ca dependent, highly thermostable and detergent compatible purified alkaline serine protease from *Nocardiopsis xinjiangensis* strain OM-6. Int J Biol Macromol.

[51] Glanville TD, Ahn H, Akdeniz N, Crawford BP, Koziel JA (2016). Performance of a plastic-wrapped composting system for biosecure emergency disposal of disease-related swine mortalities. Waste Manag.

[52] Mechri S, Berrouina MBE, Benmrad MO, Jaouadi NZ, Rekik H, Moujehed E, Chebbi A, Sayadi S, Chamkha M, Bejar S (2017). Characterization of a novel protease from *Aeribacillus pallidus* strain VP3 with potential biotechnological interest. Int J Biol Macromol.

[53] Matkawala F, Nighojkar S, Kumar A, Nighojkar A (2019). A novel thiol-dependent serine protease from *Neocosmospora* sp. N1. Heliyon.

[54] da Silva OS, de Almeida EM, de Melo AH, Porto TS (2018). Purification and characterization of a novel extracellular serine-protease with collagenolytic activity from *Aspergillus tamarii* URM4634. Int J Biol Macromol.

[55] Sinha R, Khare SK (2013). Characterization of detergent compatible protease of a halophilic *Bacillus* sp. EMB9: differential role of metal ions in stability and activity. Bioresour Technol.

[56] Giri P, Tang X, Thangamani S, Shenoy R, Ding J, Swaminathan K, Sivaraman J (2010). Modifying the substrate specificity of *Carcinoscorpius rotundicauda* serine protease inhibitor domain 1 to target thrombin. PLoS ONE.

