# Supplementary material for: Development of a microbial protease for composting swine carcasses, optimization of its production and elucidation of its catalytic hydrolysis mechanism
Source: BMC Biotechnol. 2022 Nov 28;22:36. doi: 10.1186/s12896-022-00768-0 (PMC9703648; doi:10.1186/s12896-022-00768-0)
Supplement: Supplementary file 1 — Additional file 1: Data generated in research. [file 12896_2022_768_MOESM1_ESM.docx]

**Table S1.** Physical and chemical of the compost material.

| Raw materials | Total carbon (%) | Total nitrogen (%) | Moisture content (%) |
| --- | --- | --- | --- |
| Swine carcass (carcass only) | 19.40 | 1.51 | 70.00 |
| Sawdust | 48.66 | 0.15 | 0.01 |

**Table S2.** Changes of physical and chemical properties during composting process.

| Time (d) | Temperature (℃) | C/N | pH | Moisture content (%) |
| --- | --- | --- | --- | --- |
| 0 | 29.20 | 35.92 | 6.97 | 66.75 |
| 10 | 56.30 | 22.01 | 9.10 | 56.86 |
| 20 | 43.50 | 15.46 | 9.27 | 53.82 |
| 30 | 28.20 | 19.58 | 8.21 | 49.24 |
| 40 | 26.00 | 19.88 | 8.17 | 38.22 |

* The materials were mixed (carcass/sawdust=1:1) and adjusted according to the C/N (0d) and moisture content (0d). After that, 10kg materials were put into foam box (0.39m×0.28m×0.54m) for static composting.

**Table S3.** Casein medium primary screening results. (A) FL1D. (B) ST7. (C) S12. (D) DB1. (E) DB2.

| Strain | Ratio |
| --- | --- |
| FL1D. | 1.79±0.06 |
| ST7. | 1.9±0.23 |
| S12 | 1.73±0.16 |
| DB1 | 3.93±0.27 |
| DB2 | 3.24±0.18 |

*Ratio: ratio of hydrolysis circle diameter to strain diameter.

**Table S4.** Peptide information.

| Unused | %Cov(95) | Accession | Mw(kD) | Conf | Sequence | dMass | Prec MW | Prec m/z | Theor MW | Theor m/z | Theor z |
| --- | --- | --- | --- | --- | --- | --- | --- | --- | --- | --- | --- |
| 45.56 | 29.80999947 | K7X482 | 112.922 | 99.00000095 | EAYNKGELSVNPGYGNK | 0.00155165 | 1838.88147 | 920.448 | 1838.879883 | 920.4472046 | 2 |
| 45.56 | 29.80999947 | K7X482 | 112.922 | 99.00000095 | EELIQSAAR | 0.00201037 | 1015.53186 | 508.7732 | 1015.529846 | 508.7721863 | 2 |
| 45.56 | 29.80999947 | K7X482 | 112.922 | 99.00000095 | FENANPSYPFVK | 3.59208E-06 | 1411.677246 | 706.8459 | 1411.677246 | 706.8458862 | 2 |
| 45.56 | 29.80999947 | K7X482 | 112.922 | 99.00000095 | FPYMSADQISAVIK | 0.00153354 | 1584.787354 | 529.2697 | 1584.785767 | 529.2692261 | 3 |
| 45.56 | 29.80999947 | K7X482 | 112.922 | 99.00000095 | GSQYNVEVADNGRSDK | -0.00301979 | 1738.772827 | 870.3937 | 1738.775879 | 870.3952026 | 2 |
| 45.56 | 29.80999947 | K7X482 | 112.922 | 99.00000095 | LDNGSYNFSYDK | -0.00094879 | 1422.593018 | 712.3038 | 1422.593994 | 712.3042603 | 2 |
| 45.56 | 29.80999947 | K7X482 | 112.922 | 99.00000095 | LESNTGGAVNR | -0.0019681 | 1117.534424 | 559.7745 | 1117.536377 | 559.7754517 | 2 |
| 45.56 | 29.80999947 | K7X482 | 112.922 | 99.00000095 | NGREELIQSAAR | -4.64926E-05 | 1343.679321 | 672.8469 | 1343.679321 | 672.8469238 | 2 |
| 45.56 | 29.80999947 | K7X482 | 112.922 | 99.00000095 | QDNMSFGDHGTHVAGIAAAK | -0.00215177 | 2024.898926 | 675.9736 | 2024.901001 | 675.9743042 | 3 |
| 45.56 | 29.80999947 | K7X482 | 112.922 | 99.00000095 | SGTSMAAPHVTGVAAVLMQR | -0.000396244 | 1998.997559 | 667.3398 | 1998.997925 | 667.3399048 | 3 |
| 45.56 | 29.80999947 | K7X482 | 112.922 | 99.00000095 | SQNLLSQNEAQSLLGNK | -0.00107609 | 1842.942505 | 922.4785 | 1842.943604 | 922.4790649 | 2 |
| 45.56 | 29.80999947 | K7X482 | 112.922 | 99.00000095 | TGAGTLALLGNNTYR | -5.41573E-05 | 1520.794678 | 761.4046 | 1520.794678 | 761.4046021 | 2 |
| 45.56 | 29.80999947 | K7X482 | 112.922 | 99.00000095 | VALDYQGNDVGLGITR | -0.000583121 | 1689.868042 | 845.9413 | 1689.868652 | 845.9415894 | 2 |
| 45.56 | 29.80999947 | K7X482 | 112.922 | 99.00000095 | VINNSWGIAPDIR | 0.000514896 | 1454.752319 | 728.3834 | 1454.751831 | 728.3831787 | 2 |
| 45.56 | 29.80999947 | K7X482 | 112.922 | 99.00000095 | YTILTTTDGVNGR | 3.31695E-06 | 1409.715088 | 705.8648 | 1409.715088 | 705.8648071 | 2 |
| 45.56 | 29.80999947 | K7X482 | 112.922 | 98.73999953 | NEVIAEMMR | 0.000853579 | 1091.51123 | 546.7629 | 1091.510376 | 546.7624512 | 2 |
| 45.56 | 29.80999947 | K7X482 | 112.922 | 99.00000095 | QDNMSFGDHGTHVAGIAAAKR | 0.000239515 | 2181.002441 | 546.2579 | 2181.002197 | 546.2578125 | 4 |
| 45.56 | 29.80999947 | K7X482 | 112.922 | 99.00000095 | NVEVADNGR | 0.00100227 | 972.463501 | 487.239 | 972.4624634 | 487.2385254 | 2 |
| 45.56 | 29.80999947 | K7X482 | 112.922 | 98.57000113 | VNLRDAINGPK | 0.000378076 | 1195.667725 | 598.8411 | 1195.667358 | 598.8409424 | 2 |
| 45.56 | 29.80999947 | K7X482 | 112.922 | 98.3099997 | RAFLNGGSVNVSLER | 0.000937732 | 1617.859619 | 540.2938 | 1617.858643 | 540.2935181 | 3 |
| 45.56 | 29.80999947 | K7X482 | 112.922 | 97.63000011 | AFLNGGSVNVSLER | 7.2328E-05 | 1461.75769 | 731.8861 | 1461.757568 | 731.8860474 | 2 |
| 45.56 | 29.80999947 | K7X482 | 112.922 | 97.14999795 | SKSSVEWGSEQPVPTGGHSAMSTLLR | -0.000353416 | 2743.323242 | 686.8381 | 2743.323486 | 686.8381348 | 4 |
| 45.56 | 29.80999947 | K7X482 | 112.922 | 99.00000095 | DGAGMHGVAFDADIIGTK | 0.0115027 | 1801.842041 | 601.6213 | 1801.830444 | 601.6174316 | 3 |
| 45.56 | 29.80999947 | K7X482 | 112.922 | 99.00000095 | AISAEFAYAR | 0.00307008 | 1079.543091 | 540.7788 | 1079.540039 | 540.7772827 | 2 |
| 45.56 | 29.80999947 | K7X482 | 112.922 | 99.00000095 | PDSWK | 0.000468846 | 631.2970581 | 632.3043 | 631.2965698 | 632.303833 | 1 |
| 45.56 | 29.80999947 | K7X482 | 112.922 | 99.00000095 | DISGHGGLTK | -0.000583476 | 983.5030518 | 492.7588 | 983.5036011 | 492.7590942 | 2 |
| 45.56 | 29.80999947 | K7X482 | 112.922 | 99.00000095 | GSQYNVEVADNGR | -0.00282728 | 1390.608521 | 696.3115 | 1390.611328 | 696.3129272 | 2 |
| 45.56 | 29.80999947 | K7X482 | 112.922 | 99.00000095 | AISAEFAYAR | 7.3754E-05 | 1097.550659 | 549.7826 | 1097.550537 | 549.7825317 | 2 |
| 45.56 | 29.80999947 | K7X482 | 112.922 | 99.00000095 | AISAEFAYAR | -0.000170387 | 1097.550415 | 549.7825 | 1097.550537 | 549.7825317 | 2 |
| 45.56 | 29.80999947 | K7X482 | 112.922 | 99.00000095 | FENANPSYPFVK | 0.0132538 | 1393.679932 | 697.8472 | 1393.666626 | 697.8405762 | 2 |
| 45.56 | 29.80999947 | K7X482 | 112.922 | 99.00000095 | FPYMSADQISAVIK | 0.000814414 | 1584.786621 | 793.4006 | 1584.785767 | 793.4001465 | 2 |
| 45.56 | 29.80999947 | K7X482 | 112.922 | 99.00000095 | GSQYNVEVADNGR | 0.000395193 | 1409.606323 | 705.8104 | 1409.605957 | 705.8102417 | 2 |
| 45.56 | 29.80999947 | K7X482 | 112.922 | 99.00000095 | GSQYNVEVADNGR | 0.000489268 | 1435.633301 | 718.8239 | 1435.632813 | 718.8236694 | 2 |
| 45.56 | 29.80999947 | K7X482 | 112.922 | 99.00000095 | GSQYNVEVADNGR | -0.00166642 | 1407.63623 | 704.8254 | 1407.637817 | 704.8262329 | 2 |
| 45.56 | 29.80999947 | K7X482 | 112.922 | 99.00000095 | LDNGSYNFSYDK | -1.153530002 | 1420.456421 | 711.2355 | 1421.609863 | 711.8122559 | 2 |
| 45.56 | 29.80999947 | K7X482 | 112.922 | 99.00000095 | LDNGSYNFSYDK | -0.000704649 | 1422.593262 | 712.3039 | 1422.593994 | 712.3042603 | 2 |
| 45.56 | 29.80999947 | K7X482 | 112.922 | 98.39000106 | LDNGSYNFSYDK | -1.153530002 | 1420.456421 | 711.2355 | 1421.609863 | 711.8122559 | 2 |
| 45.56 | 29.80999947 | K7X482 | 112.922 | 96.66000009 | LDNGSYNFSYDK | -0.00081993 | 1421.609131 | 711.8118 | 1421.609863 | 711.8122559 | 2 |
| 45.56 | 29.80999947 | K7X482 | 112.922 | 99.00000095 | LESNTGGAVNR | -0.000171827 | 1144.547119 | 573.2808 | 1144.547241 | 573.2808838 | 2 |
| 45.56 | 29.80999947 | K7X482 | 112.922 | 96.5200007 | NEVIAEMMR | 0.000282152 | 1123.500488 | 562.7575 | 1123.500122 | 562.7573853 | 2 |
| 45.56 | 29.80999947 | K7X482 | 112.922 | 99.00000095 | NGREELIQSAAR | -0.00114513 | 1343.678223 | 672.8464 | 1343.679321 | 672.8469238 | 2 |
| 45.56 | 29.80999947 | K7X482 | 112.922 | 99.00000095 | NGREELIQSAAR | -0.000541283 | 1342.694824 | 448.5722 | 1342.695313 | 448.5723877 | 3 |
| 45.56 | 29.80999947 | K7X482 | 112.922 | 99.00000095 | QDNMSFGDHGTHVAGIAAAKR | 0.00146022 | 2181.003662 | 546.2582 | 2181.002197 | 546.2578125 | 4 |
| 45.56 | 29.80999947 | K7X482 | 112.922 | 95.63000202 | RAFLNGGSVNVSLER | 0.000930943 | 1618.843628 | 540.6218 | 1618.842773 | 540.621521 | 3 |
| 45.56 | 29.80999947 | K7X482 | 112.922 | 99.00000095 | SGTSMAAPHVTGVAAVLMQR | 0.00371257 | 2014.996582 | 672.6728 | 2014.992798 | 672.6715698 | 3 |
| 45.56 | 29.80999947 | K7X482 | 112.922 | 99.00000095 | SGTSMAAPHVTGVAAVLMQR | 0.000959203 | 2015.977783 | 672.9999 | 2015.976807 | 672.9995728 | 3 |
| 45.56 | 29.80999947 | K7X482 | 112.922 | 99.00000095 | SGTSMAAPHVTGVAAVLMQR | -0.00243251 | 2030.985352 | 678.0024 | 2030.987793 | 678.0031738 | 3 |
| 45.56 | 29.80999947 | K7X482 | 112.922 | 99.00000095 | SQNLLSQNEAQSLLGNK | -0.000106319 | 1843.92749 | 922.971 | 1843.927612 | 922.9710693 | 2 |
| 45.56 | 29.80999947 | K7X482 | 112.922 | 97.35999703 | SQNLLSQNEAQSLLGNK | 0.000266681 | 1842.943848 | 922.4792 | 1842.943604 | 922.4790649 | 2 |
| 45.56 | 29.80999947 | K7X482 | 112.922 | 99.00000095 | TGAGTLALLGNNTYR | -0.000671298 | 1521.778076 | 761.8963 | 1521.778687 | 761.8966064 | 2 |
| 45.56 | 29.80999947 | K7X482 | 112.922 | 99.00000095 | VALDYQGNDVGLGITR | 0.000630793 | 1690.853271 | 846.4339 | 1690.852661 | 846.4335938 | 2 |
| 45.56 | 29.80999947 | K7X482 | 112.922 | 99.00000095 | VINNSWGIAPDIR | -0.00151459 | 1485.756104 | 743.8853 | 1485.757568 | 743.8860474 | 2 |
| 45.56 | 29.80999947 | K7X482 | 112.922 | 99.00000095 | VINNSWGIAPDIR | 0.000358042 | 1457.763062 | 729.8888 | 1457.762695 | 729.8886108 | 2 |
| 45.56 | 29.80999947 | K7X482 | 112.922 | 99.00000095 | VINNSWGIAPDIR | -0.00025231 | 1481.762451 | 741.8885 | 1481.762695 | 741.8886108 | 2 |
| 45.56 | 29.80999947 | K7X482 | 112.922 | 99.00000095 | VINNSWGIAPDIR | 0.000724253 | 1469.763428 | 735.889 | 1469.762695 | 735.8886108 | 2 |
| 45.56 | 29.80999947 | K7X482 | 112.922 | 99.00000095 | VINNSWGIAPDIR | -0.114395 | 1458.632324 | 730.3234 | 1458.746704 | 730.3806152 | 2 |
| 45.56 | 29.80999947 | K7X482 | 112.922 | 99.00000095 | VINNSWGIAPDIR | 0.000514896 | 1454.752319 | 728.3834 | 1454.751831 | 728.3831787 | 2 |
| 45.56 | 29.80999947 | K7X482 | 112.922 | 99.00000095 | YTILTTTDGVNGR | -0.000369683 | 1410.69873 | 706.3566 | 1410.699097 | 706.3568115 | 2 |
| 45.56 | 29.80999947 | K7X482 | 112.922 | 99.00000095 | YTILTTTDGVNGR | -0.00201203 | 1392.686523 | 697.3505 | 1392.688477 | 697.3515015 | 2 |
| 45.56 | 29.80999947 | K7X482 | 112.922 | 99.00000095 | YTILTTTDGVNGR | -0.000369683 | 1410.69873 | 706.3566 | 1410.699097 | 706.3568115 | 2 |
| 9.69 | 14.68999982 | A0A656VJZ5 | 65.323 | 99.00000095 | GIAQGPASLTNNGPNR | -0.000156744 | 1566.774902 | 784.3947 | 1566.775024 | 784.3947754 | 2 |
| 9.69 | 14.68999982 | A0A656VJZ5 | 65.323 | 99.00000095 | GSLSPQAIR | -0.00111068 | 927.5126953 | 464.7636 | 927.5137939 | 464.7641602 | 2 |
| 9.69 | 14.68999982 | A0A656VJZ5 | 65.323 | 99.00000095 | RGIAQGPASLTNNGPNR | 0.00144108 | 1722.877563 | 575.2998 | 1722.876099 | 575.2993164 | 3 |
| 9.69 | 14.68999982 | A0A656VJZ5 | 65.323 | 97.71999717 | IGNLYYAR | 0.00030845 | 968.5083008 | 485.2614 | 968.5079956 | 485.261261 | 2 |
| 9.69 | 14.68999982 | A0A656VJZ5 | 65.323 | 99.00000095 | TIHDILVNPGSGNNAVR | 1.004989982 | 1776.932861 | 889.4737 | 1775.927856 | 888.9711914 | 2 |
| 9.69 | 14.68999982 | A0A656VJZ5 | 65.323 | 99.00000095 | MNNMISVNNISELR | 0.000143036 | 1666.765625 | 834.3901 | 1666.765503 | 834.3900146 | 2 |
| 9.69 | 14.68999982 | A0A656VJZ5 | 65.323 | 96.1499989 | STGQSNITPVQISNVNFATANGPK | -0.000120153 | 2445.213379 | 816.0784 | 2445.213623 | 816.0784912 | 3 |
| 9.69 | 14.68999982 | A0A656VJZ5 | 65.323 | 98.51999879 | MNNMISVNNISELR | 0.016336801 | 1682.776733 | 842.3956 | 1682.760376 | 842.3874512 | 2 |
| 8.07 | 1.71399992 | A0A656VDJ2 | 211.911 | 99.00000095 | HVVTTLSAGK | -0.000275948 | 1011.571045 | 506.7928 | 1011.571289 | 506.7929382 | 2 |
| 8.07 | 1.71399992 | A0A656VDJ2 | 211.911 | 99.00000095 | NLTASANNQLLTYGAK | 2.72301E-05 | 1677.868652 | 839.9416 | 1677.868652 | 839.9415894 | 2 |
| 8.07 | 1.71399992 | A0A656VDJ2 | 211.911 | 99.00000095 | PAEIISGK | 0.000437866 | 813.460083 | 407.7373 | 813.4595947 | 407.7370911 | 2 |
| 6.09 | 7.191000134 | A0A656VJQ1 | 65.386 | 99.00000095 | ETELSDAQVR | -0.026860701 | 1128.514282 | 565.2644 | 1128.541138 | 565.277832 | 2 |
| 6.09 | 7.191000134 | A0A656VJQ1 | 65.386 | 99.00000095 | TGHLSEADIDASVER | 0.00042113 | 1598.754028 | 533.9253 | 1598.753662 | 533.9251709 | 3 |
| 6.09 | 7.191000134 | A0A656VJQ1 | 65.386 | 95.09000182 | GIANVMAQEGYQNVVAAK | 0.000456816 | 1877.93103 | 939.9728 | 1877.930542 | 939.9725342 | 2 |

*Unused:Protein score; %Cov(95): Proportion of peptides with reliability greater than 95%; Accession: Login number of protein in the database; Mw(kD): Theoretical molecular weight of protein; Conf: The credibility of peptide; Sequence: Peptide sequence; dMass: Difference between actual molecular weight and theoretical molecular weight of peptide segment; Prec MW: Measured molecular weight; Prec m/z: Measured m/z; Theor MW: Theoretical molecular weight of peptide; Theor m/z: Theoretical m/z; Theor z: Charge number.


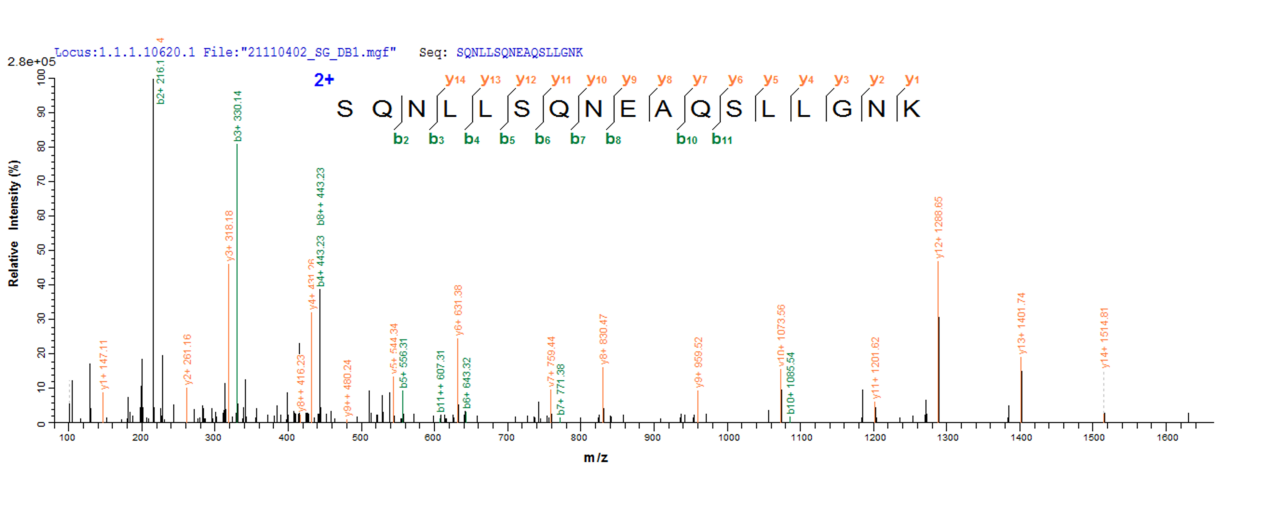


**Fig. S1.** Secondary mass spectra of one peptide of purified protease.


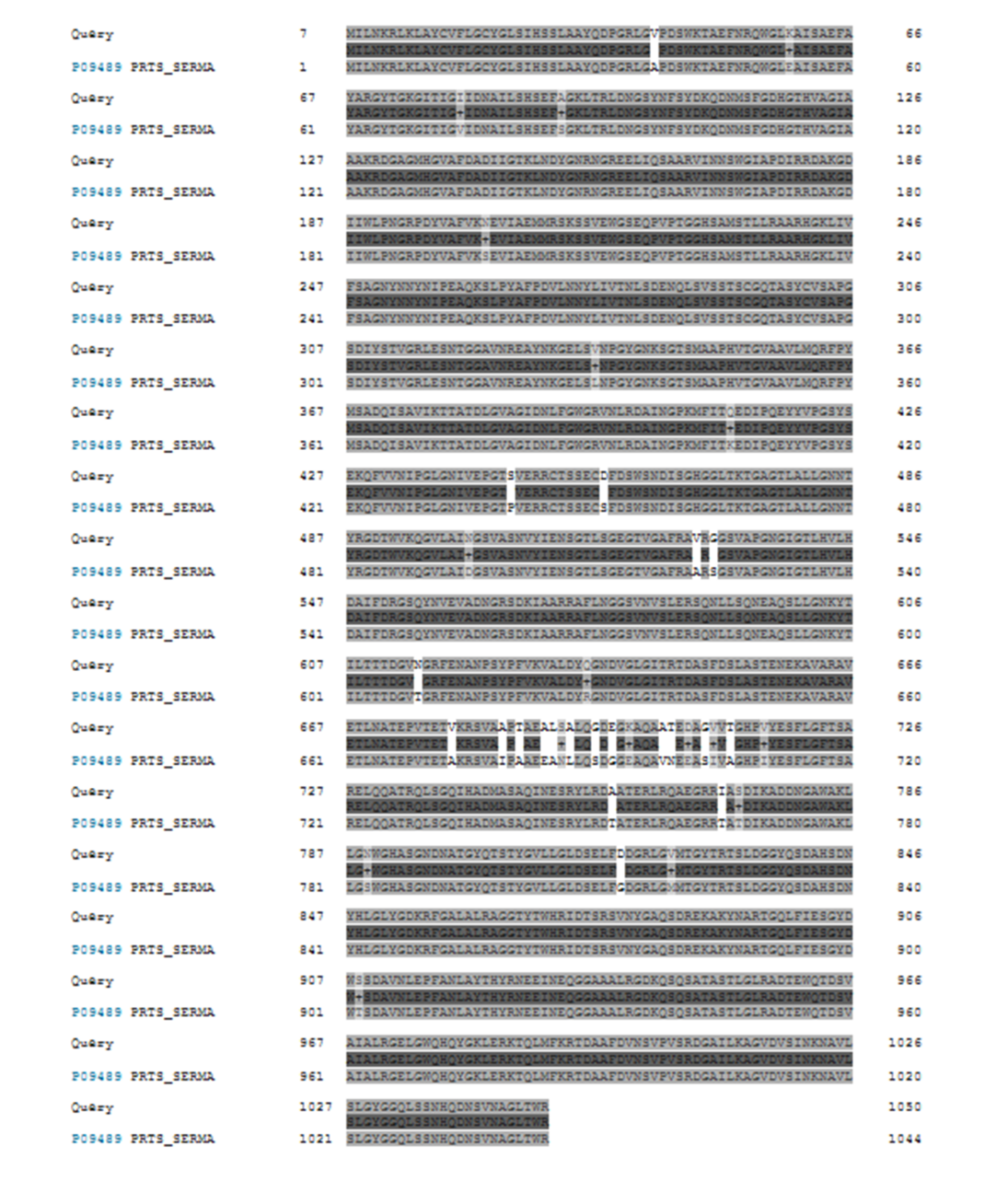


**Fig. S2.** Homology analysis of amino acid sequences of purified protease and P09489.


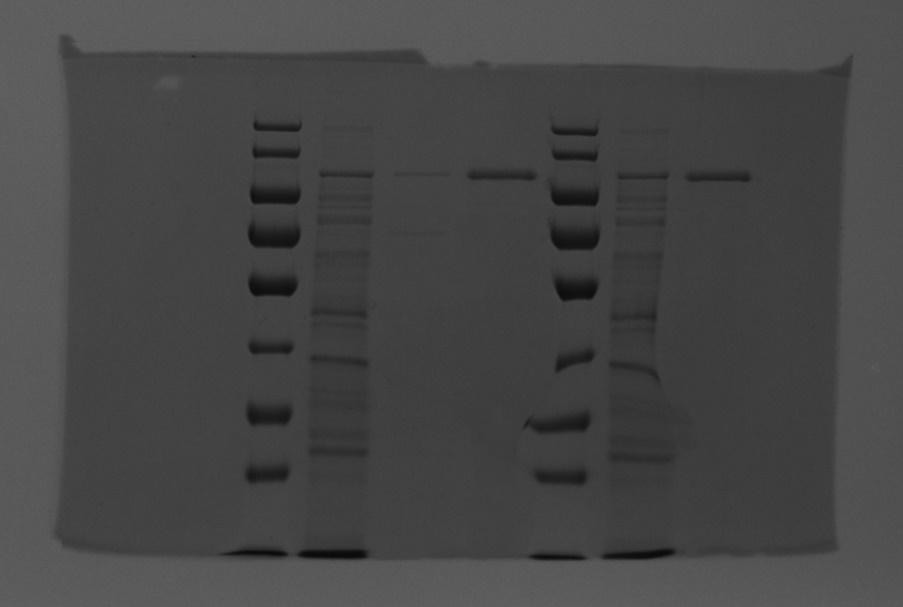


**Fig. S3.** SDS-PAGE results of proteases at various purification steps (original and unprocessed versions).

From left to right: Marker; Culture supernatant; (NH4)_2_SO_4_ precipitation (20%~40%); Sephadex G-75; Marker; Culture supernatant; Sephadex G-75.


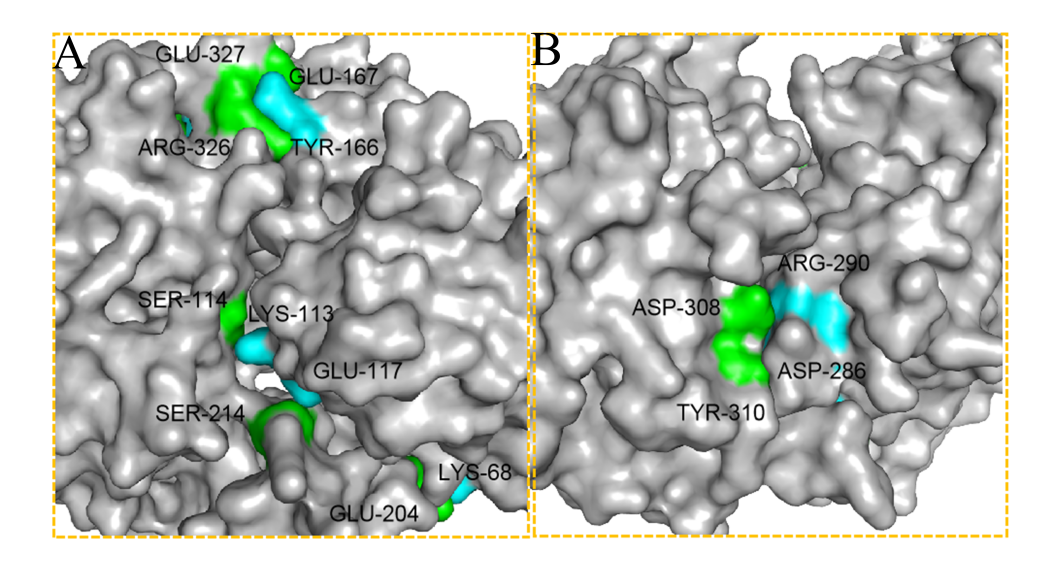


**Fig. S4.** Surface map of amino acid interaction between two proteins.
